# Research on hemodynamic state prediction based on feature-enhanced multi-model ensemble

**DOI:** 10.1016/j.mex.2026.103985

**Published:** 2026-06-02

**Authors:** Shuai Yan, Minghua Liu, Xiaoyan Wang

**Affiliations:** School of Electrical Engineering, Northwest Minzu University, Lanzhou Gansu, China

**Keywords:** Hemodynamic state prediction, Feature enhancement, Stacking ensemble, Gradient boosting trees, BOLD-fMRI

## Abstract

This study tackles the challenge of accurately inverting neural and vasodilatory signals from BOLD-fMRI data. We propose a novel framework that substantially enhances prediction performance through:

• Multi-scale dynamic feature extraction to comprehensively characterize BOLD signal properties.
• A stacking ensemble architecture that synergistically combines multiple heterogeneous base learners.
• Hierarchical model fusion via a meta-learner to robustly integrate predictions and capture complex nonlinear mappings.

Evaluated on synthetic data from the Balloon model, our method achieves an R² of 0.92 for the vasodilatory signal and 0.78 for the neural drive signal, outperforming existing benchmarks.Validation on real fMRI data shows successful reconstruction of neural activity, with reconstructed BOLD signals correlating with measured signals at levels up to 0.9931. This provides a new pathway for high-fidelity inversion of microscopic neural activity.

## Graphical abstract

**Figure:**
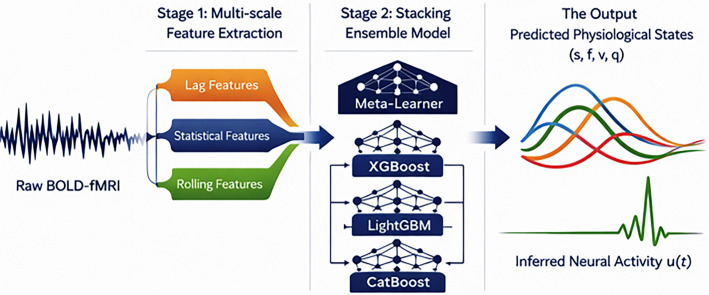

## Specifications table

**Table:** 

**Subject area**	Engineering
**More specific subject area**	Hemodynamics
**Name of your method**	Feature-Enhanced Multi-Model Stacking Ensemble Framework for Hemodynamic State Prediction
**Name and reference of original method**	Wang, X., Zhou, L., He, M., Peng, Z., & Zhang, J. (2025). XGboost with multi-feature fusion for hemodynamic state prediction. Neuroscience, 591, 63–75. https://doi.org/10.1016/j.neuroscience.2025.10.054
**Resource availability**	Data will be available on request

## Background

Functional Magnetic Resonance Imaging (fMRI), by detecting the Blood Oxygen Level Dependent (BOLD) signal, provides a non-invasive window into observing brain neural activity [1]. The BOLD signal results from a complex, nonlinear hemodynamic cascade initiated by neural activity, involving changes in local cerebral blood flow (CBF), cerebral blood volume (CBV), and deoxyhemoglobin (dHb) concentration. Therefore, accurately inferring the underlying physiological state variables, particularly the neural activity u(t), from the observed BOLD signal is crucial for understanding neurovascular coupling and its role in brain function and disorders [2,3]. However, this inverse problem is highly challenging because the BOLD signal being an indirect, noisy observation with a nonlinear, time-varying relationship to its underlying causes.

Various methodological approaches have been developed to address this challenge. Early foundational work relied on parameterized physiological models like the Balloon-Windkessel model [1]. To enhance flexibility and rigor, advanced frameworks were introduced, such as Volterra kernels [4], Bayesian inference [5], non-parametric methods [6], and nonlinear state estimation algorithms like particle filters [7] and unscented Kalman filters [8]. Havlicek et al. [9] further combined dynamic causal modeling with a Kalman filter framework.

Recently, machine learning has offered new data-driven pathways. Ensemble algorithms like XGBoost [10] have shown strong performance, while neural networks, including NARX [11] and stacked recurrent neural networks [12], have demonstrated potential. However, purely data-driven methods often act as "black-box" models with limited interpretability, and their performance heavily depends on feature representation and hyperparameter tuning. Many studies lack systematic feature engineering tailored to fMRI's unique dynamics, such as temporal delays and multi-scale fluctuations.

Consequently, current research strives to integrate model-driven and data-driven paradigms. At the feature level, systematic construction is emphasized; for instance, Wang et al. [13] confirmed that multi-dimensional features improve prediction accuracy for key states using XGBoost. Emerging techniques like Graph Neural Networks [14] and attention mechanisms [15] are also being explored. Despite progress, challenges remain in establishing standardized feature frameworks and developing ensemble strategies that synergistically integrate multiple models.

This methodology article builds upon related research, such as the work by Wang et al. [13] which utilized an XGBoost model with multi-feature fusion. The methodology described here provides a structured framework that details a comprehensive feature engineering strategy for BOLD signal dynamics and introduces a stacking ensemble protocol that integrates multiple gradient boosting models. This approach is motivated by the need to enhance predictive robustness and accuracy, particularly for challenging inverse problems like estimating neural activity u(t) from BOLD signals, by leveraging the complementary strengths of heterogeneous learners.

## Method details

### Generation of simulated data for u(t), s, f, v, q

To obtain sufficient labeled training data, this study generated a large-scale synthetic dataset based on the Balloon model. The generation of simulated data followed the method described in the literature [13] . Specifically, the neural activity sequence u(t) was simulated using an event-related design, where the neural response corresponding to each discrete stimulus was modeled as a Gaussian function. The complete u(t) sequence was generated by superimposing the responses of multiple random stimuli. The number of stimuli, their onset times, and intensities were randomly sampled from preset uniform distributions to reflect the diversity of experimental scenarios.

The hemodynamic states and BOLD signal were generated using u(t) as input, integrated numerically through the Balloon model (using a local linearization method) to obtain the continuous state variables s(t), f(t), v(t), q(t), and the corresponding BOLD signal y(t). Model parameters were set to typical values commonly used in the literature. To approximate real fMRI measurement conditions, Gaussian white noise was added to the generated BOLD signal.

**Fig. 1:**
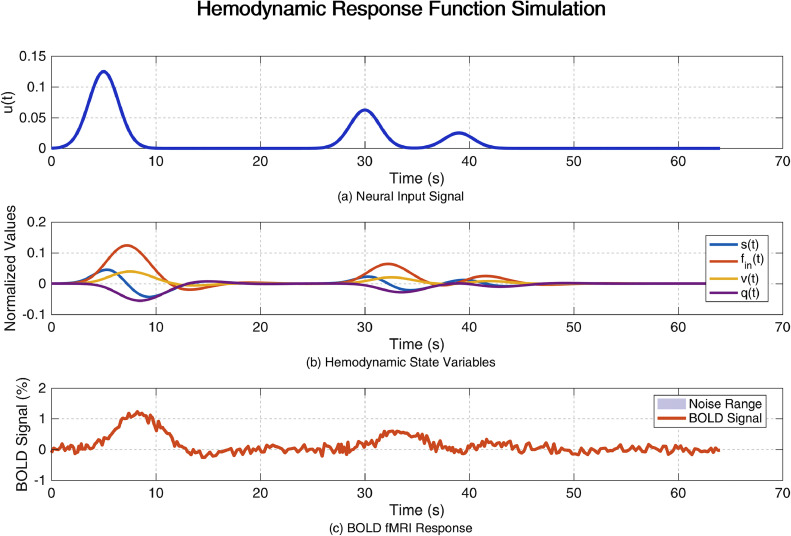
Hemodynamic response function simulation.

The dataset construction generated over 300,000 independent time series samples, each containing 64 time points. Samples were randomly split into training, validation, and test sets in a 6:2:2 ratio, with the validation set used for model hyperparameter tuning and the test set used for final performance evaluation. The simulation of the hemodynamic response function is shown in Fig. 1.

(a) Neural Input Signal (b) Hemodynamic State Variables (c) BOLD fMRI Response

### Correlation analysis of u(t), s, f, v, q with the BOLD signal

To gain an in-depth understanding of the intrinsic relationships between the BOLD signal and the underlying physiological state variables, and to provide a theoretical basis for subsequent modeling, this study first conducted a comprehensive correlation analysis on key variables in the synthetic dataset. This analysis aimed to quantitatively characterize the linear and monotonic relationships between the BOLD signal (y(t)) and its driving neural activity (u(t)), as well as the intermediate hemodynamic state variables: the vasodilatory signal (s), cerebral blood flow (f), cerebral blood volume (v), and deoxyhemoglobin concentration (q).

The results, presented in Table 1, reveal associations of varying strengths and patterns between the BOLD signal and each variable, providing important physiological insights for constructing prediction models. The BOLD signal shows an almost perfect negative correlation with deoxyhemoglobin concentration (q) (Pearson r = -0.992, R² = 0.984), directly confirming the physical basis of BOLD-fMRI, namely that its signal intensity primarily originates from local magnetic field changes induced by susceptibility differences between oxy- and deoxyhemoglobin. The BOLD signal exhibits a strong positive linear correlation with cerebral blood flow (f) and cerebral blood volume (v) (Pearson r > 0.952, R² > 0.907), reflecting that neural activity-induced increases in blood flow and volume are leading to the BOLD signal rise.

**Table 1:** Correlation analysis between state variables and the BOLD signal.

Variable	Pearson r	Pearson p	Spearman r	Spearman p	R²	Correlation Strength
s	-0.706	< 0.001	0.019	0.716	0.498	Strong
f	0.953	< 0.001	0.510	< 0.001	0.908	Extremely strong
v	0.953	< 0.001	0.240	< 0.001	0.908	Extremely strong
q	-0.992	< 0.001	-0.945	< 0.001	0.984	Extremely strong
u(t)	-0.191	< 0.001	-0.324	< 0.001	0.037	Extremely weak

The neural activity u(t), of greatest clinical and research value, shows an extremely weak correlation with the BOLD signal (Pearson r = -0.191, R² = 0.037). This result intuitively indicates that indirectly and accurately inverting its neural driver from the BOLD signal, which is confounded by multiple physiological processes and noise, is a highly ill-posed inverse problem. Also noteworthy is the vasodilatory signals, which shows a strong linear negative correlation with the BOLD signal (Pearson r = -0.706, R² = 0.498), but the Spearman rank correlation coefficient is nearly zero and not significant (r = 0.019, p = 0.716). This "divergence" phenomenon, where linear correlation is strong but monotonic correlation is weak, suggests that the dynamic coupling betweens and the BOLD signal may be complex and non-monotonic, rather than a simple increasing or decreasing trend, increasing the difficulty of accurate modeling.

The work of Wang Xiaoyan et al. confirmed that using an XGBoost model with multi-dimensional feature fusion can significantly improve prediction accuracy for states like s and f. However, the aforementioned correlation analysis results, especially the extremely weak correlation between u(t) and the BOLD signal (R² = 0.037), and the complex dynamic pattern of the s signal showing "strong linear correlation but weak monotonicity," sharply point out the inherent limitations of single gradient boosting tree models like XGBoost when facing such highly nonlinear, information-scarce, and complex-mapping inverse problems. Relying solely on a single model, its representational capacity and generalization robustness may face bottlenecks, which is particularly prominent when attempting to invert the most physiologically significant neural activity u(t).

### Feature extraction for hemodynamic state prediction model

Addressing the non-stationary, nonlinear dynamic characteristics of Blood Oxygen Level Dependent functional Magnetic Resonance Imaging time series, this study designed and implemented a systematic feature engineering framework. This framework takes the raw BOLD signal as the sole input and aims to comprehensively characterize the complex dynamic patterns of the neurovascular coupling process by constructing a multi-scale, multi-dimensional feature representation system, providing information-rich and structured input features for subsequent multi-model ensemble learning.

The feature extraction strategy of this study focuses on the temporal dynamic characteristics of the BOLD signal, avoiding reliance on frequency-domain transformations and complex feature selection algorithms in favor of a comprehensive and direct feature construction scheme. Specifically, the feature extraction process is completed through a self-implemented create_features function, which systematically generates three major categories of features from the raw BOLD signal, collectively constituting the feature space for subsequent modeling.

The first category is lag features. The study set a wide range of lag orders from 1 to 30, generating a total of 30 features: lag_1 to lag_30. This design is based on the inherent delay property of the hemodynamic response function, aiming to provide the prediction model with sufficient temporal context information, enabling it to capture typical delay and overshoot phenomena occurring approximately 6–12 time points after the response peak.

The second category is multi-scale sliding window statistical features. The study defined seven sliding windows of different scales (3, 5, 8, 10, 15, 20, 30 time points), calculating the mean and standard deviation for each window, thereby producing 14 statistical features. Among these, statistics from smaller windows (e.g., 3, 5 time points) are adept at capturing rapid fluctuations and instantaneous responses of the signal; statistics from larger windows (e.g., 20, 30 time points) are more suitable for characterizing trend changes and slow modulation processes of the signal. Such features effectively smooth random noise through local aggregation while preserving the essential dynamics of the signal at different time scales.

The third category is differencing features. By performing 1st- to 4th-order differencing operations differencing operations on the raw signal, four features are obtained: diff_1 to diff_4. The differencing operation essentially computes discrete derivatives, explicitly extracting information such as the signal's rate of change (first-order difference) and acceleration (higher-order differences), thereby enhancing the model's sensitivity to key dynamic events like turning points and response onset times.

In summary, for each input time series sample, this study constructs a total of 48 raw features. The specific feature system of the hemodynamic state inversion model is shown in Table 2, and all features are based on the historical or derived information of the BOLD signal, forming a multi-level description system from microscopic changes to macroscopic trends. All features are subsequently standardized using StandardScaler for Z-score normalization to eliminate scale differences.

**Table 2:** Feature system for hemodynamic state inversion model.

Feature Category	Feature Name Examples	Calculation Method & Parameters	Physiological Meaning
Raw BOLD Feature	BOLD	Extracted directly from the dataset	Preserves the original morphology and overall dynamics of the signal.
Lag Features	lag_1,lag_2, …, lag_30	Lag operation on the BOLD signal	Provides memory for the time series, reflecting historical dependence.
Sliding Statistical Features	rolling_mean_3, rolling_std_5, rolling_mean_15, rolling_std_30	Calculate statistics within sliding windows; window sizes: 3,5,8,10,15,20,30 time points	Characterizes local dynamic properties, smooths noise while preserving trends.
Differencing Features	diff_1,diff_2, diff_3, diff_4	1st to 4th-order differencing on the BOLD signal	Captures change trends and acceleration, enhances sensitivity to turning points.
Standardized Features	All features	Z-score standardization using StandardScaler	Eliminates scale differences, ensures fair weighting of features.

### Ensemble model construction and implementation

Addressing the extremely weak correlation (R² 0.037) between the BOLD signal and neural activity u(t) and the complex dynamic pattern of "strong linear correlation but weak monotonicity" between the vasodilatory signal s and the BOLD signal, as revealed in the aforementioned correlation analysis, single gradient boosting tree models face bottlenecks in representational capacity and generalization robustness when capturing such highly nonlinear, information-scarce, and complex-mapping inverse problems. To overcome this limitation, this study introduces a Stacked Generalization (Stacking) ensemble learning framework. Ensemble learning combines predictions from multiple base learners to achieve superior generalization performance compared to a single learner. Stacked Generalization (Stacking) is an advanced ensemble technique with a two-layer structure: the first layer (base learner layer) contains multiple heterogeneous or homogeneous base learners; the second layer (meta-learner layer) trains a meta-learner based on the prediction outputs from the first layer models to learn how to optimally combine these predictions.

This study selects three gradient boosting tree models—XGBoost, LightGBM, and CatBoost—as the base learners for the Stacking ensemble. This choice is based on their complementary design philosophies and performance advantages: XGBoost is more rigorous in regularization control and second-order approximation of the loss function, often achieving higher prediction accuracy; LightGBM employs a histogram-based algorithm and leaf-wise growth strategy, offering extremely high training efficiency and memory-friendliness, suitable for processing large-scale data with many features; CatBoost effectively mitigates gradient bias through an ordered boosting strategy, is more robust in handling categorical features, and its models often exhibit good robustness in noisy environments. The organic combination of the three aims to integrate the accuracy advantage of XGBoost, the efficiency advantage of LightGBM, and the robustness advantage of CatBoost, thereby learning the complex nonlinear mapping from BOLD signals to various state variables more comprehensively and robustly.

The Stacking ensemble framework adopted in this study is constructed through a rigorous cross-validation process, aiming to generate unbiased meta-features to enhance model generalization capability. The specific workflow is as follows:

First, multi-scale feature extraction and standardization are performed on the input BOLD signal. Following the feature system shown in Table 2, 48-dimensional features are extracted, including raw signal, lag features (lag_1 to lag_30), sliding window statistical features (e.g., rolling_mean_5), and differencing features (diff_1 to diff_4), followed by Z-score standardization.

In the base learner training and meta-feature generation stage, a five-fold cross-validation method is used. The training set is randomly divided into five subsets. Each subset is sequentially used as a validation set, while the remaining four subsets are used to independently train the three base learners: XGBoost, LightGBM, and CatBoost. Each base learner makes predictions on the validation set, and the arithmetic mean of their prediction results is taken as the meta-feature for the samples in that validation set. After completing five rounds of cross-validation, the meta-features generated from each fold are concatenated in the original sample order to form a meta-feature vector consistent with the size of the training set.

Subsequently, this meta-feature vector is used as input features, with the original hemodynamic state variables (s, f, v, q) as output labels, to train the meta-learner. This study selects Ridge regression as the meta-learner. Its simple structure combined with L2 regularization can effectively prevent overfitting in the second layer while smoothly learning the combination weights for the prediction results of the base learners.

In the final model construction stage, all three base learners are retrained using the entire training set. When making predictions on new samples, the same feature engineering and standardization are first applied. Preliminary prediction results are obtained through the base learners, averaged, and then fed into the trained Ridge regression meta-learner to obtain the final predicted values for the state variables. This workflow effectively avoids data leakage, ensuring the robustness and predictive reliability of the ensemble model.

Model performance is comprehensively evaluated using Mean Squared Error (MSE), Root Mean Squared Error (RMSE), Mean Absolute Error (MAE), and the Coefficient of Determination (R²). Furthermore, by analyzing the feature importance scores provided by the multi-model framework, we can gain an in-depth understanding of the contribution of different features to predicting each hemodynamic state, enhancing the model's interpretability.

## Method validation

### Model hyperparameter configuration

The hyperparameters of each base learner were tuned on an independent validation set using Bayesian optimization to obtain their best independent performance. The final configurations are as follows:

XGBoost: The number of trees (n_estimators) was set to 1200, the learning rate (learning_rate) to 0.02, and the maximum tree depth (max_depth) to 10. This configuration aims to balance model complexity and generalization capability. A shallower tree depth combined with a moderate number of trees helps prevent overfitting while ensuring sufficient model capacity to learn nonlinear patterns in the data.

LightGBM: Key parameters were aligned with XGBoost: number of trees is 1200, learning rate is 0.02. Model complexity is controlled by setting the number of leaves (num_leaves) to 256, ensuring the model can capture sufficient data features while maintaining efficient training.

CatBoost: The number of iterations (iterations) was set to 1500, learning rate (learning_rate) to 0.02, tree depth (depth) to 10, and an L2 regularization term was set to control overfitting. CatBoost's inherent ordered boosting mechanism and default tendency for strong regularization allow it to maintain good generalization performance under these parameter settings.

All models used a fixed random seed (random_state or random_seed = 42) to ensure experiment reproducibility, and employed Root Mean Squared Error (RMSE) and Mean Absolute Error (MAE) as evaluation metrics during training to comprehensively monitor model fitting.

### Ensemble model prediction performance

The Stacking ensemble model achieved excellent comprehensive prediction performance on the independent test set, as shown in Table 3. The prediction R² for all hemodynamic state variables exceeded 0.92. The prediction accuracy for deoxyhemoglobin content (q) was the highest (R²=0.99725), consistent with the theory that the BOLD signal is highly sensitive to changes in q. The most challenging task of neural activity (u) inversion also achieved an R² of 0.78486, significantly outperforming traditional methods.

**Table 3:** Prediction performance of the Stacking ensemble model on the test set.

Target Variable	R²	RMSE	MAE	Physiological Meaning
**Cerebral Blood Flow (f)**	**0.98309**	**0.004215**	**0.001408**	**Local blood supply.**
**Cerebral Blood Volume (v)**	**0.98850**	**0.001096**	**0.000584**	**Degree of cerebrovascular dilation.**
**Deoxyhemoglobin (q)**	**0.99725**	**0.000766**	**0.000488**	**Directly influences BOLD signal.**
**Vasodilatory Signal (s)**	**0.92038**	**0.004401**	**0.002104**	**Reflects the vasodilatory state of vascular smooth muscle.**
**Neural Activity (u)**	**0.78486**	**0.013478**	**0.002694**	**Driver of hemodynamic changes.**

**Fig. 2:**
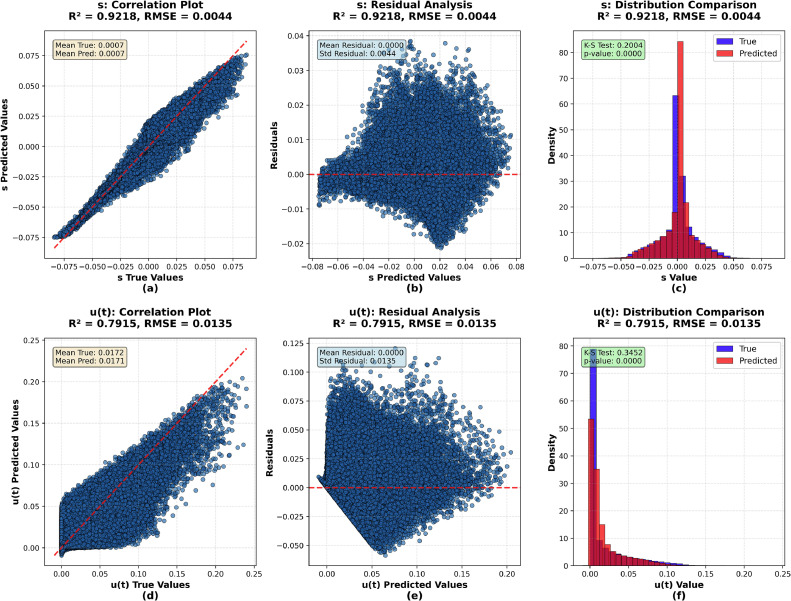
Comparison of prediction results for the vasodilatory signals and u(t).

To evaluate the model's fitting effect, we conducted residual analysis for the prediction results of s and u(t), as shown in Fig. 2. The analysis covers true vs. predicted scatter plots, residual vs. predicted plots, and distribution comparison plots. For s, the R² between true and predicted values reached 0.9204 with an RMSE of only 0.0044. The means of true and predicted values were essentially identical, with the residual mean approaching 0 and a standard deviation of 0.0044. Residuals were randomly scattered around zero without showing trend changes as predicted values increased. Additionally, the distributions of true and predicted values matched well, reflecting high fitting accuracy overall. Residuals satisfied basic assumptions such as unbiasedness and homoscedasticity, indicating strong statistical reliability of the prediction results. For u(t), the R² between true and predicted values was 0.7849 with an RMSE of 0.0135. The means of true and predicted values were close, and the residual mean also approached 0 with a standard deviation of 0.0135. Residuals were randomly distributed across the range of predicted values without obvious trend deviation. The distributions of true and predicted values also showed a certain degree of matching. Its residuals likewise conformed to the basic model assumptions, demonstrating effective fitting results and statistical reliability of the predictions.

Each row corresponds to one hemodynamic parameter (s; u(t)). Columns from left to right show: Q-Q Plot: Quantile-Quantile plot to assess normality of residuals. Residual vs Predicted (RVP) Plot: Scatter plot of residuals against predicted values to detect systematic errors. Residual Distribution:Histogram of prediction errors, with skewness and standard deviation values labeled.

### Feature importance analysis

To gain an in-depth understanding of the decision-making mechanism of the Stacking ensemble framework, this study analyzed the key features relied upon by the multi-model framework when predicting different hemodynamic state variables. Feature importance scores, from a data-driven perspective, clearly reveal specific association patterns between each state variable and the multi-scale dynamic features of the BOLD signal.

The prediction of the vasodilatory signal (s) showed a strong dependence on lag features. The top two most important features were both lag features: lag_8 (0.2367) and lag_9 (0.1976), whose cumulative importance was significantly higher than other features. This result is highly consistent with the theoretical description in the Balloon model, where the vasodilatory signal is the initial link in the hemodynamic response triggered by neural activity and exhibits significant physiological delay. The model automatically identified that the BOLD signal values from historical time points 8 and 9 were most critical for predicting the current s state, providing empirical support for the hypothesis that "lag features can effectively capture the delay in vasodilatory dynamics." Additionally, the fourth-order differencing feature diff_4 also held a certain importance (0.0895), possibly used to finely characterize higher-order dynamic information of state s changes.

**Fig. 3:**
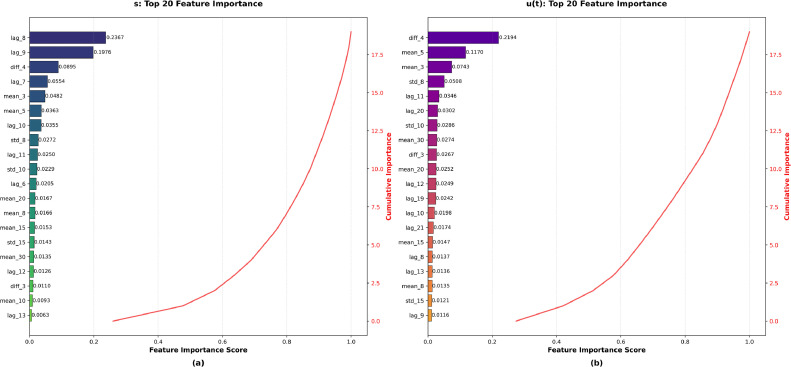
Feature importance scores for the s and u(t) prediction models.

In contrast, the feature importance pattern for neural activity u(t) exhibited significantly different characteristics. As shown in Fig. 3, its prediction was no longer dominated by a single category of features but relied on the collaborative contribution of multi-category features. Short-term sliding window statistical features, such as rolling_mean_3 and rolling_std_3, showed relatively high importance, indicating that the estimation of u(t) is closely related to the local mean and fluctuation intensity of the BOLD signal at the most recent time points. Simultaneously, first to fourth-order differencing features (diff_1 to diff_4) were also relatively prominent in importance, suggesting that capturing the instantaneous rate of change and higher-order dynamics of the BOLD signal is crucial for tracing back its neural driver. Furthermore, some lag features (e.g., lag_1, lag_2) still retained a certain contribution, but their importance was far lower than their performance in predicting s. This feature dependency pattern indicates that inverting u(t) is a more complex inverse problem, requiring a synthesis of the signal's local statistical properties, instantaneous change trends, and short-term historical information to effectively separate the weak neural driving signal from the confounded BOLD observations.

(a) Importance scores of the top twenty features for predicting the vasodilatory signal s and their cumulative importance. (b) Importance scores of the top twenty features for predicting the neural input signal u(t) and their cumulative importance.

### Comparative experiment analysis

To objectively evaluate the performance of the Stacking ensemble framework proposed in this study, we compared it with multiple models from existing research on the same synthetic dataset. The comparison models included: the high-performing gradient boosting tree model XGBoost, as well as Stacked Recurrent Neural Network (SRNN) and Nonlinear AutoRegressive with eXogenous inputs (NARX) neural network. All models used the same multi-scale feature set as input and were evaluated on an independent test set using the Coefficient of Determination (R²) as the key metric. Results are shown in Table 4.

**Table 4:** Performance comparison (R²) of Stacking ensemble and baseline models for each target variable.

model	s	f	v	q	u(t)
XGBoost	0.86	0.97	0.985	0.99	0.62
SRNN	0.842	0.963	0.978	0.955	0.632
NARX	0.85	0.97	0.974	0.99	0.60
**Stacking**	**0.92038**	**0.98309**	**0.98850**	**0.99725**	**0.78486**

The data in Table 4 shows that the Stacking ensemble framework proposed in this paper achieved optimal performance in predicting all four hemodynamic state variables. Specifically, for the most challenging task of neural activity u(t) inversion, the Stacking ensemble achieved an R² of 0.78486, significantly outperforming the best single model XGBoost (R²=0.6206), with a relative improvement exceeding 26%. This directly demonstrates that when facing the weak and highly nonlinear mapping relationship between u(t) and the BOLD signal, integrating the predictive capabilities of multiple models through a Stacking strategy can effectively overcome the representational bottleneck of a single model, achieving more accurate inversion of the neural driving signal.

For the prediction of the vasodilatory signal (s), the Stacking ensemble (R²=0.92038) also showed clear improvement compared to the best single model XGBoost (R²=0.86). For predictions of cerebral blood flow (f), cerebral blood volume (v), and deoxyhemoglobin (q), the Stacking ensemble similarly raised R² to above 0.98, with the prediction accuracy for q reaching 0.99725, nearly perfect, fully demonstrating the ensemble model's exceptional capability in capturing the strong correlation between the BOLD signal and these state variables.

Under the experimental settings of this study, the two deep learning models (SRNN, NARX) performed slightly less well overall than the XGBoost model. This is likely because the systematically constructed 48-dimensional temporal features in this study already provided rich structured information, allowing gradient boosting tree models, which excel at processing tabular data, to fully leverage their advantages. Compared to deep learning models, gradient boosting tree models typically hold advantages in training efficiency, ease of hyperparameter tuning, and result interpretability. However, even strong single models like XGBoost had their performance ceiling surpassed by the Stacking ensemble.

In summary, the comparative experiment results indicate that the proposed Stacking-based heterogeneous model ensemble framework can effectively synergize and surpass single models, including advanced gradient boosting trees and deep learning models, achieving a significant leap in prediction accuracy for hemodynamic state inversion, especially in the highly challenging task of neural activity u(t) estimation. This validates the important value of advanced ensemble learning strategies in solving complex physiological computational problems.

### Ablation experiment analysis

To systematically evaluate the specific contribution of different categories of features within the multi-scale feature engineering system constructed in this study to prediction performance, we conducted rigorous feature ablation experiments. The experiment designed seven feature combination schemes: using the full feature set (All_Features); sequentially removing all lag features (Without_Lag), removing all sliding window statistical features (Without_Rolling), removing all differencing features (Without_Diff), removing exponential moving average features (Without_EWM); and using only lag features (Lag_Only), or only rolling statistical features (Rolling_Only). All experiments were performed on the same large-scale synthetic dataset, with cross-validated average R² as the core evaluation metric. Results are summarized in Table 5.

**Table 5:** Model ablation experiment: Cross-validated average R² for each target variable under different model configurations.

model	s	u(t)
Single_XGBoost	0.8631	0.6206
Single_LightGBM	0.8632	0.6193
Single_CatBoost	0.8614	0.6077
Without_XGBoost	0.8564	0.6161
Without_LightGBM	0.8572	0.6171
Without_CatBoost	0.8600	0.6212
Simple_average	0.8641	0.6186
Weighted_average	0.8641	0.6187
**Full_Stacking**	**0.9204**	**0.78486**

In the model ablation experiment, as shown in Table 5, the full Stacking ensemble (Full_Stacking) demonstrated comprehensive superiority in cross-validation, particularly excelling in the most challenging neural activity (u) inversion task (R²=0.78486). This was significantly higher than incomplete ensembles with any one base learner removed (R² between 0.6161–0.6212), representing an improvement exceeding 26%. This indicates that XGBoost, LightGBM, and CatBoost all contribute positively to the ensemble framework and possess complementary prediction patterns. Even when the performance difference between single models is small (e.g., R² difference for s state between Single_XGBoost and Single_LightGBM is only 0.0001), their integration can still yield significant performance gains.

Further analysis of each base learner's contribution: After removing XGBoost, the R² for s state dropped to 0.8564 and for u state to 0.6161, the worst-performing combination among the three incomplete ensembles, indicating that XGBoost's high-accuracy characteristic provides the core predictive foundation for the ensemble framework. After removing LightGBM, the R² for s and u states were 0.8572 and 0.6171, respectively, slightly better than the configuration without XGBoost, suggesting LightGBM's efficient feature capture ability provides some support for model robustness. After removing CatBoost, the R² for s state was 0.8600 and for u state 0.6212, the best-performing combination among incomplete ensembles. This aligns with CatBoost's relatively weaker single-model performance. However, this configuration still fell far short of the full ensemble, confirming that while CatBoost's contribution is limited, it provides unique heterogeneous information that can compensate for prediction biases of the other two models.

More critical evidence comes from the performance comparison on the independent test set: The Stacking ensemble achieved R²=0.78486 for u(t) inversion, a 26.5% improvement compared to the best single model (XGBoost, R²=0.6206). Its RMSE and MAE were also significantly reduced to 0.013478 and 0.002694, representing reductions exceeding 22% and 74%, respectively. For s state prediction, the full ensemble's R²=0.9204 represented a 6.04 percentage point improvement over the highest value among the three incomplete ensembles (0.8600). This fully demonstrates that the nonlinear fusion mechanism achieved through the meta-learner (Ridge regression) can effectively integrate the complementary advantages of different base learners, thereby achieving more accurate and robust predictive performance on unseen data.

To systematically evaluate the specific contribution of different categories of features within the multi-scale feature engineering system constructed in this study to prediction performance, we conducted rigorous feature ablation experiments. The experiment designed seven feature combination schemes: using the full feature set (All_Features); sequentially removing all lag features (Without_Lag), removing all sliding window statistical features (Without_Rolling), removing all differencing features (Without_Diff); and using only lag features (Lag_Only), or only rolling statistical features (Rolling_Only). All experiments were performed on the same large-scale synthetic dataset using five-fold cross-validation, with cross-validated average R² (cv_r2_mean) as the core evaluation metric. Results are summarized in Table 6.

**Table 6:** Feature ablation experiment: Cross-validated average R² for each target variable under different feature combination schemes.

Feature Combination	s	f	v	q	u(t)
Without_Lag	0.8539	0.9693	0.9813	0.9958	0.6157
Without_Rolling	0.8518	0.9680	0.9805	0.9956	0.6046
Without_Diff	0.8580	0.9700	0.9817	0.9959	0.6186
Without_EWM	0.8582	0.9700	0.9817	0.9959	0.6186
Lag_Only	0.8263	0.9563	0.9724	0.9933	0.5509
Rolling_Only	0.8520	0.9690	0.9811	0.9957	0.6137
**All_Features**	**0.9204**	**0.9831**	**0.9885**	**0.9973**	**0.7845**

As shown in Table 6, the full feature set (All_Features) achieved optimal or near-optimal performance in all prediction tasks, fully validating the overall effectiveness of the multi-feature category fusion strategy.

The removal of lag features (Lag) caused the most significant performance degradation for the prediction of the vasodilatory signal (s) and neural activity (u). For s, R² decreased from 0.8584 to 0.8539; for the most challenging u, R² decreased from 0.6189 to 0.6157. This result is highly consistent with the physiological mechanisms described in the Balloon model, where vascular responses exhibit significant physiological delay and neural drivers need to be indirectly inverted from historical BOLD signals. It provides strong data-driven evidence that utilizing temporal lag information to model the "memory" effect of the hemodynamic system is crucial for accurately capturing initial vasodilatory dynamics and tracing the source of neural activity.

Multi-scale sliding window statistical features (Rolling) proved to be a universal and powerful feature category. Their removal led to a general and noticeable performance decline across all five prediction tasks, with the largest performance loss in the u(t) inversion task (R² decreased by 0.0143). This highlights the irreplaceable core role of characterizing signal local trends, fluctuation intensity, and multi-scale dynamics through means and standard deviations of different time windows for comprehensively understanding the BOLD signal and subsequently predicting its underlying physiological states.

The individual removal of differencing features (Diff) had a relatively minor impact on overall performance, indicating that the rate-of-change (derivative) information they provide overlaps to some extent with other features (especially sliding statistical features). However, their presence still provided a stable contribution to predicting states like cerebral blood flow (f), suggesting that instantaneous change information remains valuable for certain specific prediction dimensions.

A key finding is that any single category of features (e.g., Lag_Only or Rolling_Only) could not achieve the performance of the full feature set. For instance, when using only lag features, the prediction R² for u(t) dropped significantly to 0.5509, indicating severe performance loss. This confirms that the multi-dimensional feature construction strategy proposed in this study, combining "temporal lag memory + multi-scale local statistics + instantaneous rate of change," provides machine learning models with a more comprehensive and discriminative representation of sequence dynamics through complementary information from different perspectives. This systematic feature enhancement is the foundation for achieving high-precision prediction, and its necessity is validated one by one in this ablation experiment.

### Real fMRI data validation

**Fig. 4:**
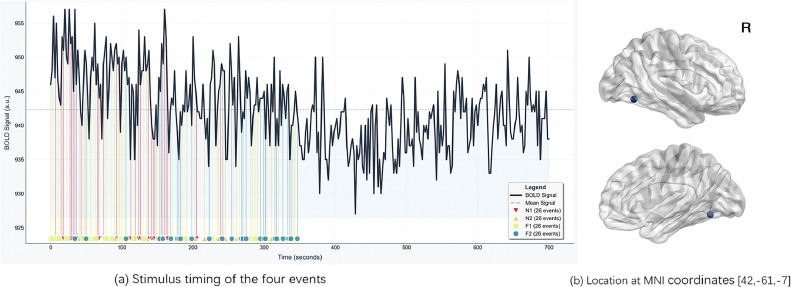
Experimental stimulus timeline and brain region localization map.

Applying the fully trained Stacking ensemble model to real-world scenarios is a crucial step to test its generalization capability and physiological plausibility. Therefore, we selected four independent validation samples from a publicly available event-related fMRI dataset for end-to-end testing. This study used the event-related fMRI dataset provided by the SPM website (http://www.fil.ion.ucl.ac.uk/spm/data/face_rep/) to validate the model's performance. This dataset examines neural response differences to repeated presentations of famous and non-famous faces. The experiment employed a 2 (face type: famous/non-famous) × 2 (presentation order: first/second) design, resulting in four conditions: F1 (famous first), F2 (famous second), N1 (non-famous first), and N2 (non-famous second). The experimental stimulus timeline and brain region localization are shown in Fig. 4.

(a)Stimulus timing of the four events. (b)Location at MNI coordinates [42,-61,-7].

**Fig. 5:**
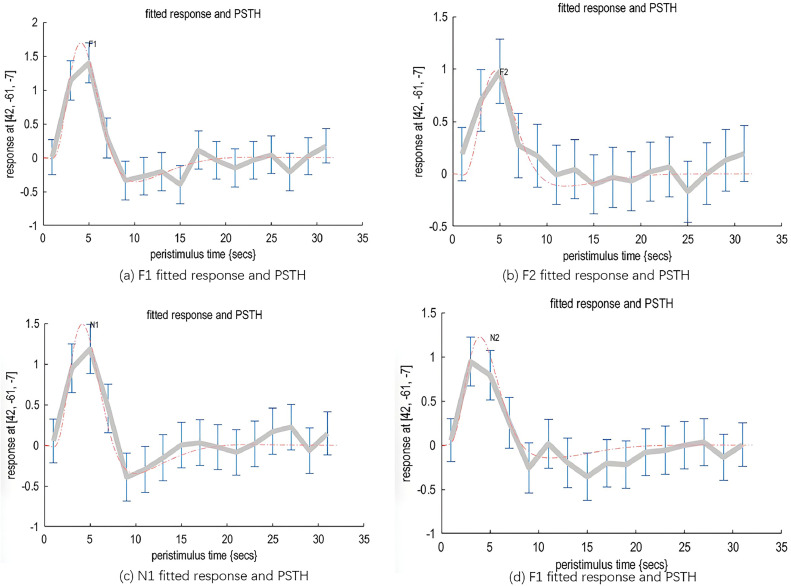
Event-related response patterns and fitted curves under each condition.

The data were preprocessed using SPM8 (including motion correction and slice timing correction), followed by standard GLM analysis procedures. Peri-stimulus time histogram (PSTH) curves for the F1, F2, N1, and N2 conditions were extracted from the right fusiform face area, and the actual BOLD signal was fitted using an HRF and its temporal derivative model. The event-related response patterns and fitted curves for each condition are shown in Fig. 5. These reflect the temporal dynamic effects of different stimulus types and repeated presentations on neural activation, providing the experimental basis for subsequently inverting neural activity u(t) from the BOLD signal.The fitted event-related BOLD responses were used to estimate hidden states. The complete analysis pipeline is: Perform multi-scale feature extraction on the preprocessed real BOLD signal, input it into the model to predict the dynamic curves of the vasodilatory signal s, cerebral blood flow f, cerebral blood volume v, and deoxyhemoglobin content q, and then inversely estimate the neural activity u(t) from these predicted states based on the hemodynamic model.

(a) F1 fitted response and PSTH (b) F2 fitted response and PSTH(c) N1 fitted response and PSTH(d) N2 fitted response and PSTH

In terms of neural activity estimation, the model demonstrated a strong capability to capture temporal dynamic patterns. As shown in Fig. 7, although the signal directly output by the model and the theoretical u(t) calculated via state variables differed in amplitude scale, after simple amplitude and temporal alignment adjustments, their waveforms showed high consistency. The coefficient of determination (R²) between the adjusted estimated signal and the calculated values increased to a range of 0.6478 to 0.7774, significantly higher than the ineffective level before adjustment. More importantly, all estimated u(t) curves exhibited clear transient activation precisely at the stimulus presentation times of the experimental task, accurately matching the event-related nature of the task design. This confirms that the model can effectively separate task-evoked neural activity components from the complex BOLD signal.

**Fig. 6:**
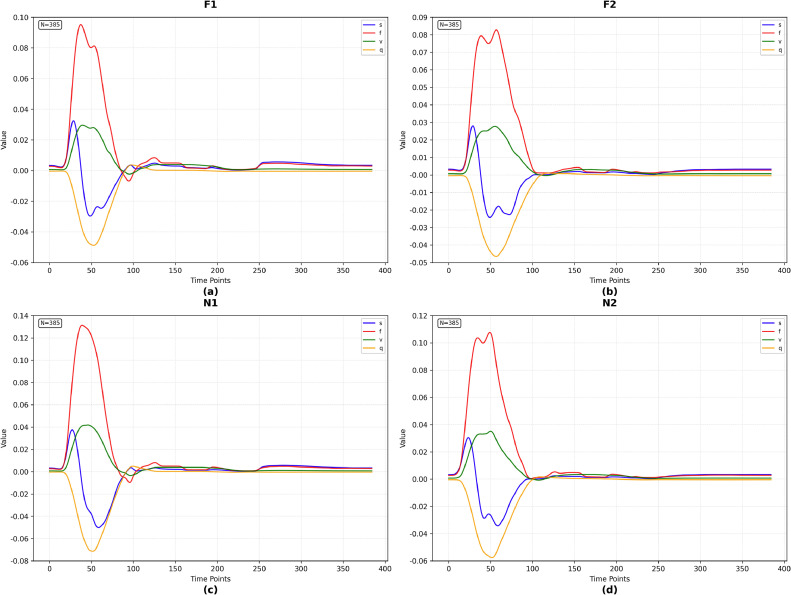
Predicted curves for s, f, v, q states based on real event-related BOLD responses.

**Fig. 7:**
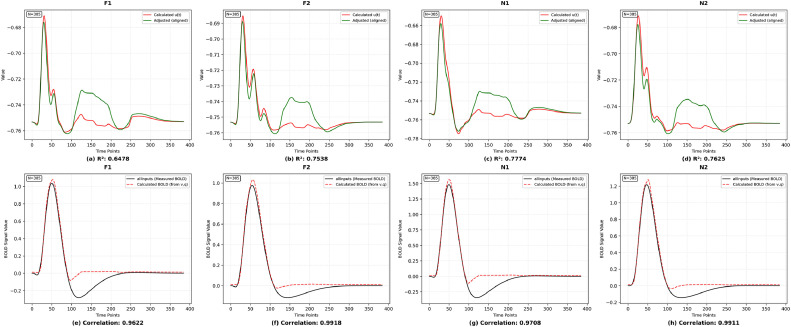
u(t) estimation results based on real event-related BOLD responses.

Another core efficacy of the model is reflected in the accurate inversion of hemodynamic state variables. As shown in Fig. 6, the model's predicted curves for s, f, v, q are smooth and exhibit reasonable physiological temporal relationships: vasodilation leads, followed by changes in cerebral blood flow and volume, ultimately reflected in the dynamic adjustment of deoxyhemoglobin concentration. The physiological plausibility of these prediction results was further validated, as shown in Fig. 7: Substituting the predicted state variables back into the BOLD signal generation equation resulted in reconstructed theoretical BOLD signals that achieved extremely high correlation with the actual observed signals, with correlation coefficients ranging from 0.9622 to 0.9911. The reconstructed signals fully reproduced the typical hemodynamic response morphology present in the real data, including the initial undershoot, main overshoot, and post-overshoot undershoot characteristics.

(a), (b), (c), and (d) are the predicted state variable time courses (s(t): vasodilation signal; f(t): cerebral blood flow; v(t): blood volume; q(t): deoxyhemoglobin content) obtained by applying the model to four real datasets (F1, F2, N1, N2), respectively. The model successfully captures the temporal evolution of each state variable.

Subfigures (a), (b), (c), and (d) present a comparison of neural drive estimates for the four real datasets (F1, F2, N1, N2). The green curve represents the directly predicted neural drive signal u(t). The red curve represents the theoretical BOLD signal calculated from the full set of predicted hemodynamic states (s, f, v, q).Subfigures (e), (f), (g), and (h) show a comparison between the measured and model-predicted BOLD signals for the same four datasets (F1, F2, N1, N2). The black curve represents the known (measured) input BOLD signal. The red curve represents the theoretical BOLD signal calculated specifically from the predicted hemodynamic state variables v and q.

These results indicate that the prediction framework constructed in this study successfully bridges the gap between synthetic and real data. The model can not only accurately recover the temporal evolution trajectories of various physiological states from noisy real BOLD signals but also inversely infer neural driving signals time-locked to cognitive events. The high correspondence between the reconstructed and measured signals, from a closed-loop validation perspective, ensures the physiological reliability of the entire inversion process. This marks that the framework has initial potential for application in actual fMRI data analysis, providing a new, powerful computational tool for in vivo studies of neurovascular coupling mechanisms.

## Discussion

Despite the remarkable predictive performance achieved by the proposed feature-enhanced multi-model ensemble framework—particularly in the challenging task of neural activity inversion—its translation into clinical practice is hindered by two major obstacles: model interpretability and integration with clinical workflows.

The Stacking ensemble strategy, by combining heterogeneous gradient boosting models, significantly boosts accuracy but at the cost of transparency. The resulting "black-box" nature makes it difficult for clinicians to understand how predictions are derived, which is a critical requirement for trust and adoption in medical decision-making. For instance, while the model successfully estimates the neural drive signal u(t) from BOLD time series, the complex interplay of multi-scale features that leads to a given prediction remains opaque. To bridge this gap, future work should incorporate explainable local interpretable models, or attention mechanisms—to quantify the contribution of individual features and provide intuitive justifications for each output. Such transparency would not only enhance clinical confidence but also facilitate the discovery of novel physiological insights.

Equally challenging is the integration of the proposed framework into routine clinical workflows. The current pipeline relies on extensive multi-scale feature extraction and post-processing steps, which are computationally demanding and ill-suited for real-time applications. In a clinical setting, speed, stability, and ease of use are paramount. Therefore, efforts should be directed toward model lightweighting.Moreover, a user-friendly interface that allows clinicians to input data, visualize predictions, and explore model reasoning without technical expertise is essential for successful adoption.

Ultimately, the transition from a research-grade tool to a clinically valuable assistant will require close collaboration between data scientists, neuroscientists, and clinicians. Prospective validation on diverse real-world datasets, along with iterative refinement based on clinical feedback, will be crucial to ensure robustness, generalizability, and practical utility in diagnosing and monitoring neurological disorders.

## Limitations

None.

## Ethics statements

This study did not involve human participant data collected through social media platforms. All data use is fully compliant with the redistribution policies of each platform. No identifiable personal information is used to ensure compliance with ethical research standards.

## CRediT authorship contribution statement

**Shuai Yan:** Conceptualization, Methodology, Investigation, Formal analysis, Visualization. **Minghua Liu:** Formal analysis, Writing – review & editing. **Xiaoyan Wang:** Conceptualization, Writing – review & editing.

## Declaration of competing interest

The authors declare that they have no known competing financial interests or personal relationships that could have appeared to influence the work reported in this paper.

## Data Availability

Data will be made available on request.

## References

[1] Buxton R B, Wong E C, Frank L R (1998). Dynamics of blood flow and oxygenation changes during brain activation: the balloon model. Magn. Reson. Med..

[2] Devi R., Lepsien J., Lorenz K., Schlumm T., Mildner T., Möller H.E. (2022). Multi-echo investigations of positive and negative CBF and concomitant BOLD changes. NeuroImage.

[3] Havlicek M., Roebroeck A., Friston K.J., Gardumi A., Ivanov D., Uludag K. (2017). On the importance of modeling fMRI transients when estimating effective connectivity: a dynamic causal modeling study using ASL data. NeuroImage.

[4] Friston K.J., Mechelli A., Turner R., Price C.J. (2000). Nonlinear responses in fMRI: the balloon model, volterra kernels, and other hemodynamics. NeuroImage.

[5] Friston K.J., Penny W., Phillips C., Kiebel S., Hinton G., Ashburner J. (2002). Classical and Bayesian inference in neuroimaging: theory. NeuroImage.

[6] Riera J.J., Watanabe J., Kazuki I., Naoki M., Aubert E., Ozaki T., Kawashima R. (2004). A state-space model of the hemodynamic approach: nonlinear filtering of BOLD signals. NeuroImage.

[7] Gordon N.J., Salmond D.J., Smith A.F.M. (1993). Novel approach to nonlinear/non-gaussian bayesian state estimation. IEE Proc. F Radar Signal Process..

[8] Julier S.J., Uhlmann J.K. (2004). Unscented filtering and nonlinear estimation. Proc. IEEE.

[9] Havlicek M., Friston K.J., Jan J., Brazdil M., Calhoun V.D. (2011). Dynamic modeling of neuronal responses in fMRI using cubature Kalman filtering. NeuroImage.

[10] T. Chen, C. Guestrin, XGBoost: A Scalable Tree Boosting System, in: Proceedings of the 22nd ACM SIGKDD International Conference on Knowledge Discovery and Data Mining, 2016, pp. 785–794, 10.1145/2939672.2939785.

[11] Karam A.M., Laleg-Kirati T.M., Zayane C., Kashou N.H. (2014). Nonlinear neural network for hemodynamic model state and input estimation using fMRI data. Biomed. Signal Process. Control.

[12] Yao Y., Junzhong J. (2018). Hemodynamic State estimation method based on stacked recurrent neural network. J. Autom. Sin..

[13] Wang X., Zhou L., He M., Peng Z., Zhang J. (2025). XGboost with multi-feature fusion for hemodynamic state prediction. Neuroscience.

[14] Hospedales T.M., Antoniou A., Micaelli P., Storkey A.J. (2022). Meta-learning in neural networks: a survey. IEEE Trans. Pattern Anal. Mach. Intell..

[15] Vaswani A., Shazeer N., Parmar N. (2017). Attention is all you need. Adv. Neural Inf. Process. Syst..

